# Short-term exposure to traffic-related air pollution and dynamic brain connectivity in adolescents

**DOI:** 10.1016/j.dcn.2025.101574

**Published:** 2025-05-27

**Authors:** Mónica López-Vicente, Michelle S.W. Kusters, Sami Petricola, Henning Tiemeier, Ryan L. Muetzel, Mònica Guxens

**Affiliations:** a ISGlobal, Barcelona, Spain; b Universitat Pompeu Fabra, Barcelona, Spain; c Spanish Consortium for Research on Epidemiology and Public Health (CIBERESP), Instituto de Salud Carlos III, Madrid, Spain; d Department of Child and Adolescent Psychiatry/Psychology, Erasmus MC, Rotterdam, the Netherlands; e Department of Social and Behavioral Sciences, Harvard T. H. Chan School of Public Health, Boston, MA, USA; f Department of Radiology and Nuclear Medicine, Erasmus MC, Rotterdam, the Netherlands; g ICREA, Barcelona, Spain

**Keywords:** Air pollution, Short-term, Magnetic Resonance Imaging, Brain, Functional connectivity, Adolescent

## Abstract

There is some evidence which suggests short-term effects of traffic-related air pollution on brain function in adults. We aimed to examine these associations at ages 10 and 14 years using dynamic functional brain connectivity. We included participants from a population-based birth cohort with brain connectivity and air pollution data at home (n = 3608) or school (n = 2305) in at least one visit. We used land use regression models to estimate levels of air pollutants, including nitrogen oxides (NO_X_) and particulate matter (PM), during the week before the outcome measurement. Using resting-state functional magnetic resonance imaging data, we generated five connectivity patterns. We calculated the mean time spent in each pattern for each participant and visit. We performed linear mixed effects models adjusted for relevant confounders. The median levels of NO_X_ at the two visits and at home and school were between 36 and 47 μg/m^3^ and the median levels of PM_2.5_ were between 11 and 12 μg/m^3^. We found a weak association between higher air pollution exposure and less time spent in a low modularized connectivity pattern (e.g. coefficient=-0.031 [95 % confidence interval=-0.056; −0.006] per 20 μg/m^3^ increase in NO_X_ at home). However, this association did not remain after multiple testing correction. Further research that explores these associations at other exposure levels and other age periods is warranted.

## Highlights

• We estimated the air pollution levels during the week before the brain scan.
• We tested associations with the time spent in different brain connectivity patterns.
• Short-term air pollution was weakly associated with the brain connectivity patterns.
• None of the tested associations survived multiple testing correction.
• We used a large sample size and we adjusted the models for multiple confounders.

## Graphical abstract

**Figure:**
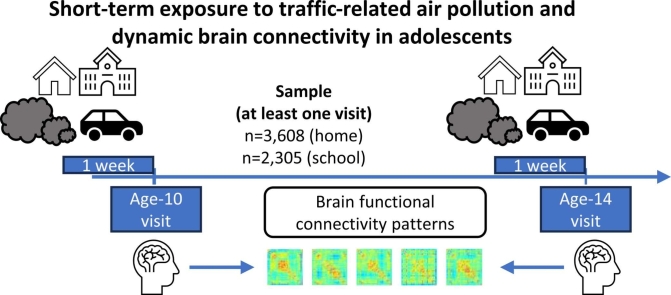

## 1 Introduction

According to the World Health Organization, air pollution is the single biggest environmental threat to human health (World Health Organization, 2021). Most studies on neurodevelopment have investigated the effects of long-term exposure to air pollution (World Health Organization, 2021, Guxens et al., 2022, Suades-González et al., 2015). At the brain functional level, higher long-term air pollution exposure has been associated with decreased brain blood flow (Peterson et al., 2022) and weaker connectivity between regions belonging to the same network (within-network connectivity) and stronger connectivity between regions from different networks (between-network connectivity) (Pérez-Crespo et al., 2022, Pujol et al., 2016). Two recent studies explored the relationship between air pollution and changes in development of brain network connectivity (Cotter et al., 2023, Kusters et al., 2025). Cotter et al. found that some pollutants were associated with stronger between-network connectivity over a 2-year follow-up during adolescence, while others showed positive associations with within-network connectivity (Cotter et al., 2023). Kusters et al. found persistent associations between air pollution and lower functional connectivity between the amygdala and the ventral attention, somatomotor hand, and auditory networks throughout adolescence (Kusters et al., 2025). The most common approach to measure brain functional connectivity is to calculate correlations between spontaneous activity in different brain regions inferred from resting-state functional magnetic resonance imaging (rs-fMRI) data. During brain development, the connectivity between regions that are part of the same network tends to increase, thus becoming more integrated (Luciana, 2013).

Short-term air pollution exposure, usually measured a few hours or days prior to the outcome assessment, has also been shown to have an impact on the central nervous system (Song et al., 2023). Specifically, short-term nitrogen dioxide (NO_2_) exposure was associated with increased serum levels of nervous system damage biomarkers in older adults. The exposure to air pollutants may cause an inflammatory response (Hu et al., 2020) and cardiovascular alterations, (Brook et al., 2010) which may, in turn, affect the brain in just few hours. In adults, short-term exposure to air pollution has been associated with brain activity changes measured with electroencephalogram (Ke et al., 2022) and with weaker functional connectivity within the default mode network, which is active during self-referential processes (Gawryluk et al., 2023). Although some studies observed short-term associations of air pollution with worse cognitive performance in children, (Saenen et al., 2016, Sunyer et al., 2017, Mizen et al., 2020) no studies are available on the short-term associations between air pollution and brain function in children or adolescents.

Therefore, this study aimed to examine the short-term associations of several traffic-related air pollutants at home and school settings with brain dynamic functional connectivity at 10 and 14 ages. Similarly to previous studies, (Pérez-Crespo et al., 2022, Pujol et al., 2016, Gawryluk et al., 2023) brain functional connectivity was measured using rs-fMRI. However, we applied a dynamic connectivity approach, which allowed us to capture different reoccurring connectivity configurations across the scanning session, instead of using the traditional static approach of computing average correlations (Allen et al., 2014, Calhoun et al., 2014). The identification of these changing connectivity configurations provides additional information about the function and development of the brain. For instance, there are specific changes in the frequency of certain connectivity configurations and the time spent in them related to age. During brain development, there is an increase in the time spent in modularized patterns, characterized by positive within-network connectivity and negative between-network connectivity (Rashid et al., Aug 2018, López-Vicente et al., 2021). Alterations in dynamic connectivity, specifically increased time spent in less modularized connectivity patterns, have been related to autism spectrum disorder and schizophrenia (Rashid et al., Aug 2018, Damaraju et al., 2014). These differences in brain function between psychiatric patients and controls are only detected using a dynamic approach (Calhoun et al., 2014). Given that previous research found that short-term exposure to air pollution was related to lower within-network connectivity (Gawryluk et al., 2023) and to worse cognitive performance, (Saenen et al., 2016, Sunyer et al., 2017) we expected to find that a higher exposure to air pollution would be related to more time spent in less modularized connectivity patterns.

## 2 Methods

### 2.1 Participants

This work is part of the Generation R Study, a population-based birth cohort in Rotterdam, the Netherlands (Kooijman et al., 2016). The original purpose of this cohort was to investigate early environmental and genetic factors related to health from fetal life until young adulthood. A total of 9901 children born between April 2002 and January 2006 were initially recruited. Three subjects were excluded because they requested data removal a posteriori. Rs-fMRI data was acquired at the age-10 years visit (8-to-12, n = 3240) (White et al., 2018), between March 2013 and November 2015, and the age-14 years visit (13-to-15, n = 2146), between October 2016 and January 2020. We excluded participants with prominent brain imaging incidental findings or low-quality data (due to excessive motion or poor registration). The flow chart is depicted in eAppendix 1. A total of 3608 participants had good quality rs-fMRI and air pollution data at home in at least one visit, while 2305 participants had good quality rs-fMRI and air pollution data at school in at least one visit. All participants provided assent (younger than 12 years) or informed consent (12 years or older). The local medical ethics committee of the Erasmus MC University Medical Center approved all study procedures.

### 2.2 Exposures

Using land use regression (LUR) models, we calculated the average exposure to five air pollutants (NO_2_, nitrogen oxides (NO_X_), particulate matter with aerodynamic diameter < 10μm (PM_10_) and < 2.5 μm (PM_2.5_), and PM_COARSE_) at residential address and schools during the week before the MRI scan at each visit (Guxens et al., 2022, Beelen et al., 2013, Eeftens et al., 2012, de Hoogh et al., 2013, Jedynska et al., 2014, Yang et al., 2015). We calculated PM_2.5_ absorbance at residence only because this data was not available at schools. Within the ESCAPE (European Study of Cohorts for Air Pollution Effects) project, several air pollutants were monitored in various seasons between February 2009 and February 2010 in the Netherlands and Belgium. NO_2_ and NO_X_ were measured at 80 sites across these countries and PM_10_ and PM_2.5_ were measured at 40 of those sites. PM_2.5_ was subtracted from PM_10_ to calculate PM_COARSE_. PM_2.5_ filters collected at the 40 sites were used to measure the absorbance of PM_2.5_ fraction. The measurements were corrected for temporal variability using data from a monitoring station and they were used to calculate annual mean concentrations for each pollutant. Next, the potential land use predictors (such as proximity to the nearest road, traffic intensity on the nearest road, and population density) that explained most of the air pollutant concentrations were assessed using LUR models and the air pollution levels were estimated for each participant residential and school addresses.

We extrapolated the concentrations to the days of the MRI scans and seven days before using the ratio method (Brunekreef, 2012). We first collected daily air pollution data for seven routine monitoring sites that covered the area of interest, as it was set in the extrapolation procedure of ESCAPE. Then, using the daily data from the monitoring sites, we calculated the annual average concentration for the period corresponding to the ESCAPE measurement campaigns. We calculated the ratio between the daily air pollution data and the annual average for the ESCAPE measurement period. The daily ratios of each monitoring site were averaged over all the monitoring sites. When data from the monitoring sites were unavailable for a given pollutant for the period of interest, we used measurements of another one instead. Specifically, we used NO_X_ when PM_2.5_ absorbance was missing and PM_10_ when PM_2.5_ was missing. The selection of the pollutant used to extrapolate the missing one was done based on the temporal correlations between pollutants that were simultaneously available. The daily ratios were then multiplied by the estimated annual mean concentrations at each participant address. Finally, we calculated the weekly averages.

### 2.3 Magnetic resonance imaging

**Fig. 1:**
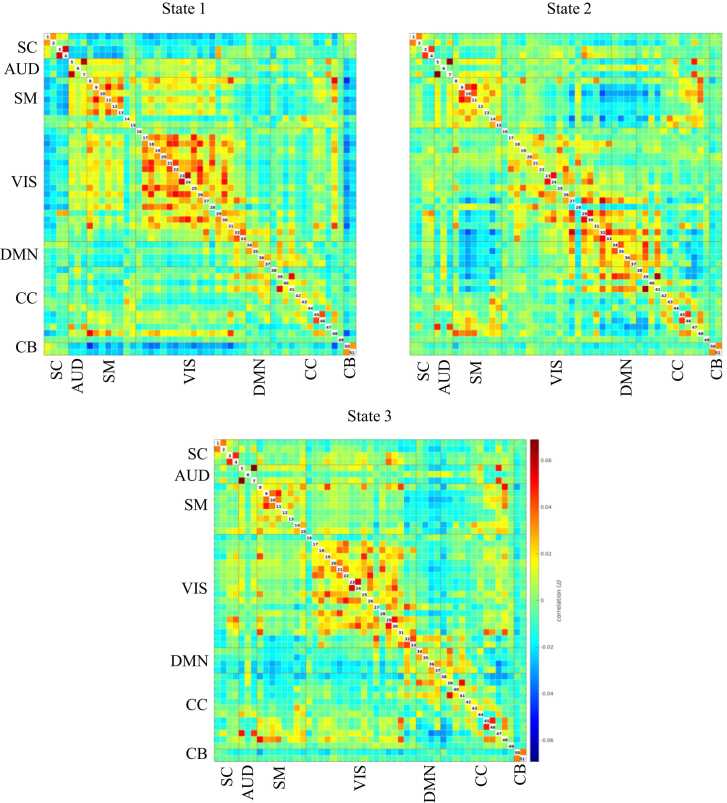
Modularized states. Abbreviations: SC, subcortical network; AUD, auditory network; SM, sensorimotor network; VIS, visual network; DMN, default-mode network; CC, cognitive control network; CB, cerebellar network.

**Fig. 2:**
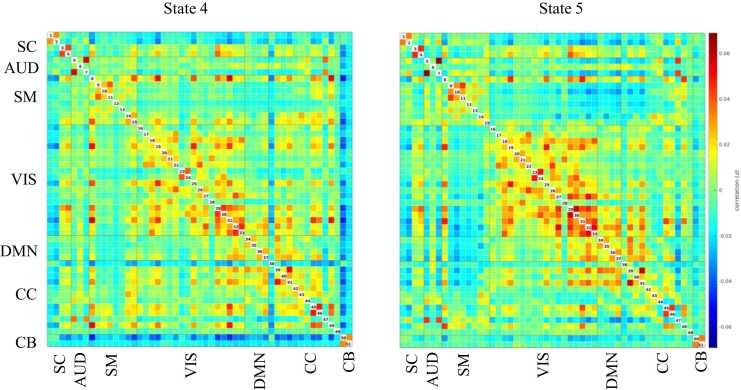
Non- and partially modularized states. Abbreviations: SC, subcortical network; AUD, auditory network; SM, sensorimotor network; VIS, visual network; DMN, default-mode network; CC, cognitive control network; CB, cerebellar network.

We used a study-dedicated 3 Tesla GE Discovery MR750w MRI System (General Electric, Milwaukee, WI, United States) scanner with an 8-channel head coil. The details of data acquisition and processing are explained in eAppendix 2. We excluded scans with excessive motion, defined as having a mean framewise displacement (FD) higher than 0.25 mm or having more than 20 % of the volumes with a FD higher than 0.2 mm. In the present study, we used dynamic connectivity data computed previously using the same sample (López-Vicente et al., 2021). Briefly, after image pre-processing, we applied a group-independent component analysis, using 51 components grouped into seven networks: subcortical, auditory, sensorimotor, visual, default-mode, cognitive control, and cerebellar (eAppendix 3). We used a tapered sliding window approach (window size of 25 TR) and k-means clustering on the time windows from each scan session to identify five patterns of connectivity, called “states”, that reoccur within the scan sessions and across subjects and visits. Three of the states were modularized, in which the components showed within- and between-network connectivity (states 1, 2, and 3, Fig. 1) and two of the states were non- or only partially modularized states (states 4 and 5, Fig. 2). State 1 was considered a “drowsy” state (Allen et al., 2014). In this state, subcortical and sensorimotor networks showed positive within-network connectivity and negative between-network connectivity. In state 2, sensorimotor and default-mode networks showed positive within-network connectivity and negative between-network connectivity. Frontal cognitive control components showed positive connectivity with default-mode network and negative connectivity with sensorimotor network, while posterior cognitive control components showed the opposite pattern. In state 3, components from the default-mode network were positively connected and they were negatively connected with components from other networks, except frontal cognitive control components. States 2 and 3 might indicate a higher awareness, either towards the outside stimuli (sensorimotor) or self-referential processes (default-mode). State 4 was non-modularized, with no modular organization of functional connectivity in distinguished networks. The putamen (subcortical , 1 and 2), the middle temporal gyrus (cognitive control, 38), and the cerebellum were negatively connected with all the other components in this state. State 5 was partially modularized, showing sub-modules within networks. For instance, the putamen (subcortical, 1 and 2) showed negative connectivity with visual components, while the thalamus (subcortical, 3 and 4) was positively connected with those components. The postcentral gyrus component (sensorimotor, 8) was positively connected with visual components, and the rest of the sensorimotor components were negatively connected with them. For each individual and visit, we obtained the mean dwell time in each dynamic state (i.e., the average time windows spent in the states). We transformed the mean dwell time outcomes using Box-Cox from the ‘bestNormalize’ R package due to their right-skewed distribution.

### 2.4 Measurement of covariates

We defined covariates a priori using a directed acyclic graph (eAppendix 4) based on scientific literature, on data availability, and on percentage of missing values (<35 %) (Kusters et al., 2025, Guxens et al., 2018). According to our theoretical framework, parental socioeconomic status would be related both to exposures and outcomes. We used multiple proxies to control as much as possible for this key confounder. The following variables were collected during pregnancy using questionnaires: maternal and paternal age and national origin, maternal education level, maternal smoking during pregnancy, maternal alcohol consumption during pregnancy, maternal folic acid supplement during pregnancy, maternal parity, marital status, and monthly household income. Maternal pre-pregnancy body mass index was calculated based on self-reported weight and measured height. Maternal IQ was assessed using the Raven Advanced Progressive Matrices Test at 6 years of child age (Raven et al., 1962).

As a measure of chronic or long-term exposure, we estimated the average air pollution values of the childhood period (from birth to the age-10 visit) for each participant using LUR models. For the participants who moved, we weighted the exposure levels by the time spent living at each address. Green space exposure was assessed using the Normalized Difference Vegetation Index (NDVI) in the surrounding area of 300 m of the home address for the pregnancy period. This index is based on satellite data that estimates the degree of land surface reflectance of light (Rhew et al., 2011). The socioeconomic status of the neighborhood during pregnancy was estimated using mean household income, and proportion of population with low income, low educational level, and without paid work, from the Dutch National Institute for Public Health and the Environment (NIPHE, 2017). Average temperature and humidity for the week before the MRI visits at home addresses were obtained from the ENSEMBLES Observations (E-OBS) gridded dataset, available for all Europe on 0,1 deg resolution (around 11 km) (Cornes et al., 2018).

### 2.5 Statistical analyses

We performed multiple imputation of missing covariate values for the two analysis samples (home air pollution sample = 3608 and school air pollution sample = 2305) using chained equations (‘mice’ R package) to generate 25 complete datasets. We performed inverse probability weighting using the Covariate Balancing Propensity Score method (‘CBPS’ R package) to correct analyses for potential attrition bias due to different characteristics between included and excluded participants (eTable 1 and eAppendix 5).

To test the associations between each pollutant (average exposure during the week before the MRI scan) and each dynamic connectivity outcome separately, we fitted linear mixed effects models using data from both MRI visits and including the participant as random effect. We performed different models for home and school settings, given the different sample size. These models allow the inclusion of repeated measures of both exposure and outcome, taking into account the within-participant correlation. We performed these analyses using the R package ‘lme4’. We checked and confirmed the linearity of the associations using the R package ‘gam’. We adjusted the main models for covariates that changed between assessments (time-varying covariates), including participant’s age at MRI assessments, temperature, humidity, season, day of the week, and time of the day when the scan was conducted. We also included stable covariates in the main models, such as season of birth, maternal IQ, maternal national origin, maternal pre-pregnancy body mass index, parental ages, maternal education, monthly household income, maternal smoking, maternal alcohol consumption, maternal folic acid supplement, residential surrounding greenness, socioeconomic status of the neighborhood, maternal parity, and marital status. All variance inflation factor (VIF) values were below 10. Moreover, we performed two secondary sets of models: i) minimally-adjusted models, which were only adjusted for time-varying covariates, and ii) models adjusted for average exposure to each specific pollutant at home from birth to the age-10 visit (long-term air pollution), in addition to the time-varying and stable covariates included in the main models. As sensitivity analyses, we also performed linear regression models separated by visit. We present all beta coefficients and their 95 % confidence intervals per 10 μg/m^3^ for NO_2_; 20 μg/m^3^ for NO_X_; 5 μg/m^3^ for PM_2.5_; 10 μg/m^3^ for PM_10_; 5 μg/m^3^ for PM_COARSE_; 10^−5^ m^−1^ for PM_2.5_absorbance, based on the distribution of each exposure variable. We estimated the effective number of tests to correct the results for multiple testing over the five outcomes (mean dwell time in each dynamic state) taking into account their non-independence, which yielded a new p-value threshold of 0.01 (Galwey, 2009). All statistical analyses were performed using the R Statistical Software (version 4.3.1).

## 3 Results

**Fig. 3:**
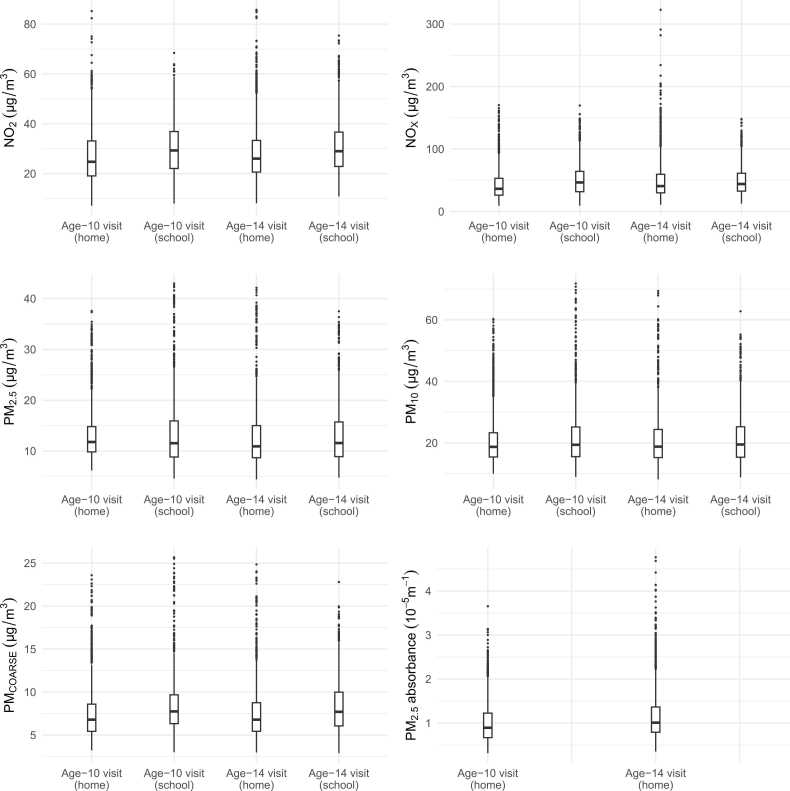
Distribution of average air pollutant levels at home and school during the week before the first and the second MRI visits. Abbreviations: NO_X_, nitrogen oxides; NO_2_, nitrogen dioxide; PM, particulate matter with different aerodynamic diameters: less than 10 μm (PM_10_); between 10 μm and 2.5 μm (PM_COARSE_); less than 2.5 μm (PM_2.5_); PM_2.5_ absorbance, absorbance of PM_2.5_ filters.

Half of the participants included in this study had mothers with high education level and the maternal national origin of 57 % of the participants was the Netherlands (Table 1). The MRI scans were acquired from Monday to Saturday, between 8:00 and 18:00 hours, and evenly distributed across the four seasons. During the week before the age-10 MRI visit days, the median NO_X_ levels were 36.39 μg/m^3^ and 46.55 μg/m^3^, and the median PM_2.5_ levels were 11.77 μg/m^3^ and 11.54 μg/m^3^ at home and school, respectively (Fig. 3). During the week before the age-14 MRI visit days, the median NO_X_ levels were 40.70 μg/m^3^ and 44.00 μg/m^3^, and the median PM_2.5_ levels were 10.91 μg/m^3^ and 11.57 μg/m^3^ at home and school, respectively. The highest correlations (> 0.75) were observed among NO_X_, NO_2_, and PM_2.5_ absorbance, and among PM_2.5_, PM_10_, and PM_COARSE_ (eAppendix 6 and 7).

**Table 1:** Sociodemographic characteristics of the study participants (n = 3608).

Variables	Categories	Distribution
Participant sex (%)	Boy	48.0
	Girl	52.0
Participant age 10-years visit (mean, SD)		10.2 (0.6)
Participant age 14-years visit (mean, SD)		14.0 (0.6)
Maternal age at intake (mean, SD)		31.0 (4.9)
Maternal pre-pregnancy body mass index (kg/m^2^) (mean, SD)		23.6 (4.1)
Maternal education level (%)	High	49.9
	Medium	42.6
	Low	7.5
Maternal national origin (%)	Netherlands	57.0
	Morocco	5.2
	Surinam	7.7
	Turkey	6.0
	Other European	7.7
	Other non-European	16.4
Marital status (%)	Married/living together	88.2
	No partner	11.8
Parity (%)	Nulliparous	56.6
	1 child	31.0
	2 + children	12.4
Monthly household income during pregnancy (%)	> 2200 euro	60.2
	1600–2200 euro	14.5
	900–1600 euro	15.9
	< 900 euro	9.4

We observed that higher exposure to NO_X_ at home was associated with less time spent in state 4, a non-modularized state with no clear organization of functional connectivity in distinguished networks (transformed mean dwell time coefficient = −0.031 [95 % CI = −0.056; −0.006] per 20 μg/m^3^ increase in NO_X_, p value = 0.02, Table 2). However, this association did not remain after multiple testing correction. The exposure to air pollution at school was not associated with dynamic functional connectivity.

**Table 2:** Short-term associations (beta and 95 % confidence interval) between exposure to each air pollutant at home (n = 3608) and school (n = 2305) and mean dwell time in the five states, not adjusted for long-term exposure.

Exposures		State 1	State 2	State 3	State 4	State 5
NO_2_ (Δ 10 μg/m^3^)	Home	−0.007 (−0.036; 0.023)	0.017 (−0.016; 0.050)	0.004 (−0.029; 0.036)	−0.027 (−0.060; 0.005)	−0.010 (−0.041; 0.021)
	School	−0.003 (−0.040; 0.034)	0.006 (−0.036; 0.047)	−0.017 (−0.058; 0.023)	−0.008 (−0.049; 0.034)	0.030 (−0.009; 0.069)
NO_X_ (Δ 20 μg/m^3^)	Home	0.009 (−0.013; 0.032)	0.018 (−0.007; 0.043)	0.005 (−0.020; 0.030)	−0.031 (−0.056; −0.006)*	−0.013 (−0.036; 0.011)
	School	−0.002 (−0.033; 0.028)	−0.006 (−0.040; 0.028)	−0.023 (−0.056; 0.011)	0.001 (−0.033; 0.035)	0.022 (−0.010; 0.054)
PM_2.5_ (Δ 5 μg/m^3^)	Home	0.002 (−0.026; 0.029)	0.005 (−0.026; 0.036)	−0.001 (−0.031; 0.030)	0.001 (−0.029; 0.031)	0.001 (−0.028; 0.030)
	School	−0.002 (−0.031; 0.028)	−0.008 (−0.042; 0.025)	−0.006 (−0.038; 0.027)	0.032 (−0.002; 0.065)	0.010 (−0.021; 0.042)
PM_10_ (Δ 10 μg/m^3^)	Home	0.003 (−0.032; 0.038)	0.016 (−0.023; 0.056)	0.008 (−0.031; 0.047)	−0.009 (−0.047; 0.030)	−0.006 (−0.043; 0.031)
	School	0.001 (−0.038; 0.040)	−0.008 (−0.052; 0.036)	−0.008 (−0.051; 0.035)	0.038 (−0.006; 0.081)	0.016 (−0.026; 0.058)
PM_COARSE_ (Δ 5 μg/m^3^)	Home	−0.002 (−0.049; 0.045)	0.017 (−0.035; 0.070)	0.013 (−0.038; 0.065)	−0.007 (−0.058; 0.045)	−0.008 (−0.058; 0.041)
	School	0.004 (−0.052; 0.060)	0.001 (−0.061; 0.064)	0.012 (−0.049; 0.073)	0.040 (−0.022; 0.103)	0.049 (−0.010; 0.108)
PM_2.5_ absorbance (10^−5^ m^−1^)	Home	0.015 (−0.049; 0.079)	0.034 (−0.038; 0.105)	−0.005 (−0.075; 0.066)	−0.069 (−0.139; 0.001)	0.004 (−0.063; 0.071)

Abbreviations: NO_X_, nitrogen oxides; NO_2_, nitrogen dioxide; PM, particulate matter with different aerodynamic diameters: less than 10 μm (PM_10_); between 10 μm and 2.5 μm (PM_COARSE_); less than 2.5 μm (PM_2.5_); PM_2.5_ absorbance, absorbance of PM_2.5_ filters. State 1, “drowsy”; State 2, default-mode/sensorimotor modularized; State 3, default-mode network modularized; State 4, non-modularized; State 5, partially modularized. Linear mixed effects models performed independently for each exposure and outcome, adjusted for participant’s age at MRI assessments, temperature, humidity, season, day of the week, time of the day when the scan was conducted, season of birth, maternal IQ, maternal national origin, maternal pre-pregnancy body mass index, and covariates during pregnancy, such as parental ages, maternal education, monthly household income, maternal smoking, maternal alcohol consumption, maternal folic acid supplement, residential surrounding greenness, socioeconomic status of the neighborhood, maternal parity, and marital status. * p value < 0.05 (non-significant after multiple testing correction, new p-value 0.01).

The minimally-adjusted models, which were only adjusted for time-varying covariates, showed the same association between exposure to NO_X_ at home and time spent in state 4 (transformed mean dwell time coefficient = −0.033 [95 % CI = −0.057; −0.008] per 20 μg/m^3^ increase in NO_X_, p value = 0.01, eAppendix 8). In addition, higher exposure to NO_2_ and PM_2.5_ absorbance was also associated with less time spent in this state. Only NO_X_ survived multiple testing correction. We did not observe any short-term associations between traffic-related air pollution and brain dynamic functional connectivity after including long-term air pollution exposure in the models (e.g., transformed mean dwell time coefficient = −0.025 [95 % CI = −0.054; 0.005] per 20 μg/m^3^ increase in NO_X_, p value = 0.10, eAppendix 9). We found these null associations both with air pollution levels at home and at school and with all the outcomes (i.e., time spent in the five states). The results of the models performed separately by visit were generally similar to the main results (eAppendix 10 and 11). We found an association between NO_2_ at school and less time spent in state 1, the “drowsy” state, only in the age-10 visit (transformed mean dwell time coefficient = −0.055 [95 % CI = −0.108; −0.003] per 10 μg/m^3^ increase in NO_2_, p value = 0.04). However, this association was not significant after multiple testing correction.

## 4 Discussion

We did not find short-term associations between exposure to traffic-related air pollutants and brain dynamic functional connectivity in adolescents. This was the first study that explored the short-term associations between these exposures and brain functional connectivity at this relevant developmental age.

Previous studies carried out in adult samples of small size found alterations in brain functional connectivity related to short-term air pollution exposure (Ke et al., 2022, Gawryluk et al., 2023). PM_2.5_ exposure was associated with alterations in brain activity measured with electroencephalogram three days after and these alterations mediated the associations between PM_2.5_ and executive functions (Ke et al., 2022). An experimental study using resting-state fMRI found that the acute exposure to diesel exhaust yielded a decrease in functional connectivity of the default-mode network compared to exposure to filtered air (Gawryluk et al., 2023). In children, short-term exposure to air pollution was inversely associated with cognitive performance, such as attention, and educational attainment (Saenen et al., 2016, Sunyer et al., 2017, Mizen et al., 2020).

A likely explanation for not finding short-term associations with dynamic connectivity in adolescents could be that the effect, if any, is small. Differences between this study and previous research include different outcomes used, different study designs, and sample characteristics (size and age). The dynamic connectivity method that we applied in this study splits the MRI measures in very short time windows and clusters the data in different connectivity patterns. This allows to capture variations in the brain functional connectivity and disentangle the brain activity from the noise signal. In addition, compared to previous research in this field, we used a whole-brain approach of functional connectivity (i.e., we focused on patterns of connectivity instead of specific networks), which could also contribute to explain the different findings. Some of the previous studies used measures of air pollution, either personal (Ke et al., 2022) or at schools (Saenen et al., 2016, Sunyer et al., 2017), instead of modelled estimations, or more repeated outcome assessments over shorter periods of time (i.e., four assessments over one year (Sunyer et al., 2017)). Regarding the experimental study (Gawryluk et al., 2023), participants were exposed to very high levels of PM_2.5_ (300 μg/m^3^) and only during 120 minutes. Thus, the exposure conditions are not entirely comparable to our study. Another experimental study with more comparable exposure levels and sample size conducted in adolescents did not find short-term associations with attention function, in line with our findings (Gignac et al., 2021). Our models were adjusted for several confounders, not only related to the outcome assessment (time-varying covariates), but also socioeconomic status. The inclusion of covariates that were stable in time weakened the associations in our study. Therefore, all these factors, alone or in combination, may play a role in explaining the differences in results observed among studies.

We used standardized and validated models to estimate the exposure levels for each participant both at home and school. However, our findings might be affected by non-differential misclassification, given that the measurement sites were limited in time and space. Also, although we included exposures both at home and school in our analyses, we were not able to combine the estimations due to differences in sample size. We did not take into account characteristics of the home and school buildings or the ventilation of the spaces where the participants spent most of their time. These variables could influence the air pollutant levels to which the participants were exposed. These limitations could have resulted in a possible underestimation of true associations. In comparison to previous research on this topic, the sample size of this study was a strength. Although we adjusted the association models for a comprehensive list of confounders, including socioeconomic covariates, there could still be residual confounding. Finally, the different characteristics of the included and excluded participants could have led to selection bias. Although we implemented inverse probability weighting to correct the analyses for this bias, we could have missed important predictors of inclusion.

In conclusion, we did not find short-term associations between air pollution and brain dynamic functional connectivity in adolescents. Further research that explores these associations at other exposure levels and other age periods is warranted.

## CRediT authorship contribution statement

**Henning Tiemeier:** Writing – review & editing, Supervision, Project administration. **Ryan L. Muetzel:** Writing – review & editing, Supervision, Software, Resources, Project administration. **Mònica Guxens:** Writing – review & editing, Supervision, Funding acquisition, Conceptualization. **Mónica López-Vicente:** Writing – original draft, Visualization, Software, Funding acquisition, Formal analysis, Data curation, Conceptualization. **Michelle S.W. Kusters:** Writing – review & editing, Validation, Investigation. **Sami Petricola:** Writing – review & editing, Software, Data curation.

## Declaration of Competing Interest

The authors declare that they have no known competing financial interests or personal relationships that could have appeared to influence the work reported in this paper.

## Data Availability

The datasets generated and analyzed during the current study are not publicly available due to legal and ethical regulations, but may be made available upon request to the director of the cohort in accordance with the local, national, and European Union regulations: Vincent Jaddoe (v.jaddoe@erasmusmc.nl).
