# Supplementary material for: Short-term exposure to traffic-related air pollution and dynamic brain connectivity in adolescents
Source: Dev Cogn Neurosci. 2025 May 27;74:101574. doi: 10.1016/j.dcn.2025.101574 (PMC12169711; doi:10.1016/j.dcn.2025.101574)
Supplement: Supplementary file 1 — Supplementary material [file mmc1.docx]

**Supplementary Material: Short-term exposure to traffic-related air pollution and dynamic brain connectivity in adolescents**

Mónica López-Vicente, Michelle Kusters, Sami Petricola, Henning Tiemeier, Ryan Muetzel, Mònica Guxens

Contents

[eAppendix 1. Flowchart of the study population 2](#_Toc196213670)

[eAppendix 2. Magnetic Resonance Imaging 3](#_Toc196213671)

[eAppendix 3. Components used for the group-independent component analysis 4](#_Toc196213672)

[eAppendix 4. Directed Acyclic Graph 5](#_Toc196213673)

[eAppendix 5. Distribution of the inverse probability weights (IPW) 6](#_Toc196213674)

[eAppendix 6. Spearman correlations between pollutant levels at home during the week before the MRI visits 7](#_Toc196213675)

[eAppendix 7. Spearman correlations between pollutant levels at school during the week before the MRI visits 8](#_Toc196213676)

[eAppendix 8. Short-term associations (beta and 95% confidence interval) between exposure to each air pollutant at home (n = 3,608) and school (n = 2,305) and mean dwell time in the 5 states, adjusted for time-varying covariates only 9](#_Toc196213677)

[eAppendix 9. Short-term associations (beta and 95% confidence interval) between exposure to each air pollutant at home (n = 3,608) and school (n = 2,305) and mean dwell time in the 5 states, adjusted for long-term exposure 10](#_Toc196213678)

[eAppendix 10. Short-term associations (beta and 95% confidence interval) between exposure to each air pollutant at home (n = 2,475) and school (n = 1,498) and mean dwell time in the 5 states, age-10 years visit 11](#_Toc196213679)

[eAppendix 11. Short-term associations (beta and 95% confidence interval) between exposure to each air pollutant at home (n = 2,121) and school (n = 1,312) and mean dwell time in the 5 states, age-14 years visit 12](#_Toc196213680)

[References 13](#_Toc196213681)


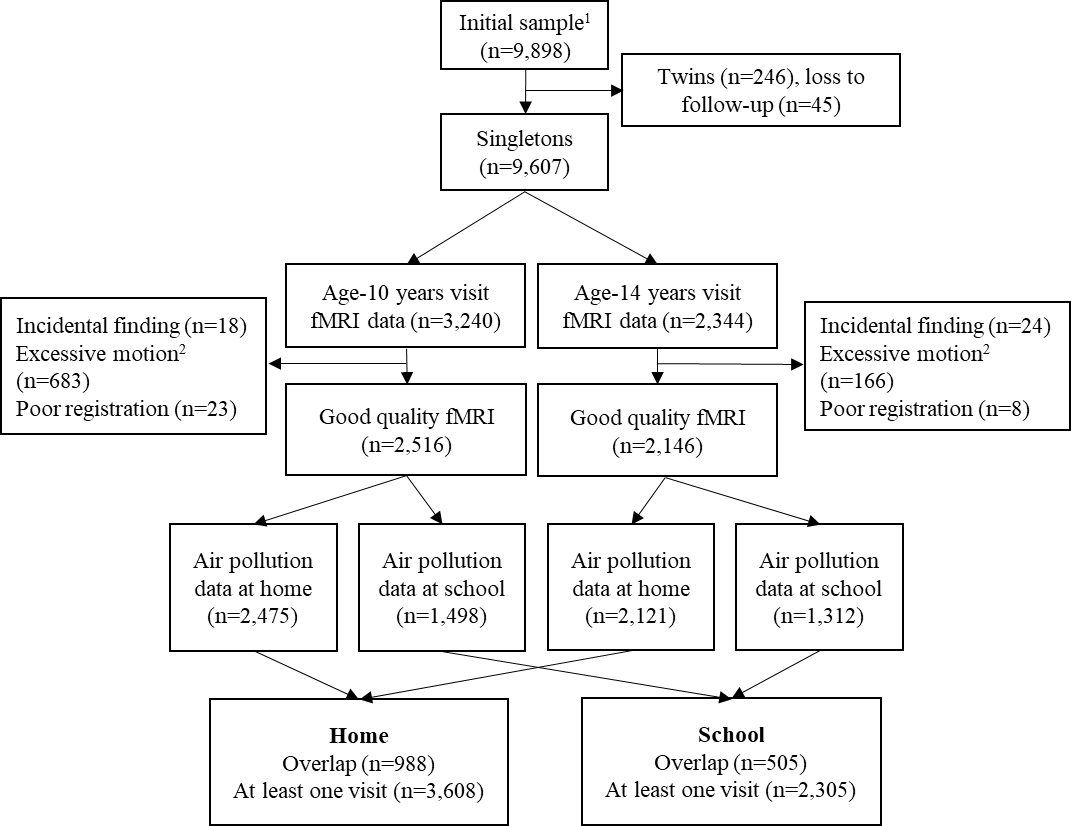


# eAppendix 1. Flowchart of the study population

^1^Initial sample excludes 3 subjects who requested data removal a posteriori.

^2^Excessive motion was defined as having a mean framewise displacement (FD) higher than 0.25 mm or having more than 20% of the volumes with a FD higher than 0.2 mm.

# eAppendix 2. Magnetic Resonance Imaging

Structural T1-weighted images were obtained using a 3D coronal inversion recovery fast spoiled gradient recalled (IR-FSPGR, BRAVO) sequence using ARC acceleration. We used an interleaved axial echo planar imaging sequence to acquire 200 volumes of resting-state functional MRI data.^1^ The resting-state scan duration was 5 min 52 s. The participants received instructions to stay awake and keep their eyes closed.

The FMRIPrep package (version 20.1.1 singularity container) was used to preprocess data.^2^ For the dynamic functional connectivity analyses, we used the Group ICA Of fMRI Toolbox (GIFT) software (GroupICAT v4.0b) in MATLAB R2020a.


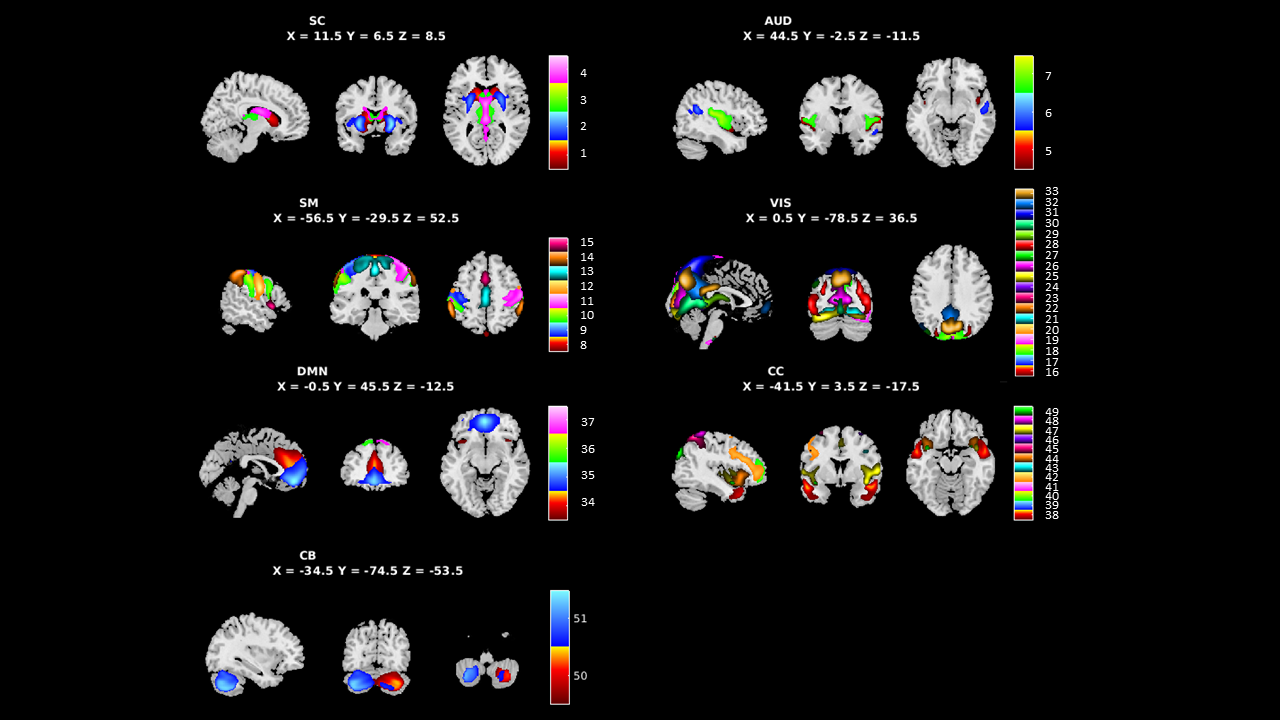


eAppendix 3. Components used for the group-independent component analysis

The 51 resting state components grouped by networks, including 4 subcortical (SC), 3 auditory (AUD), 8 sensorimotor (SM), 18 visual (VIS), 4 default-mode (DMN), 12 cognitive control (CC), and 2 cerebellar (CB).


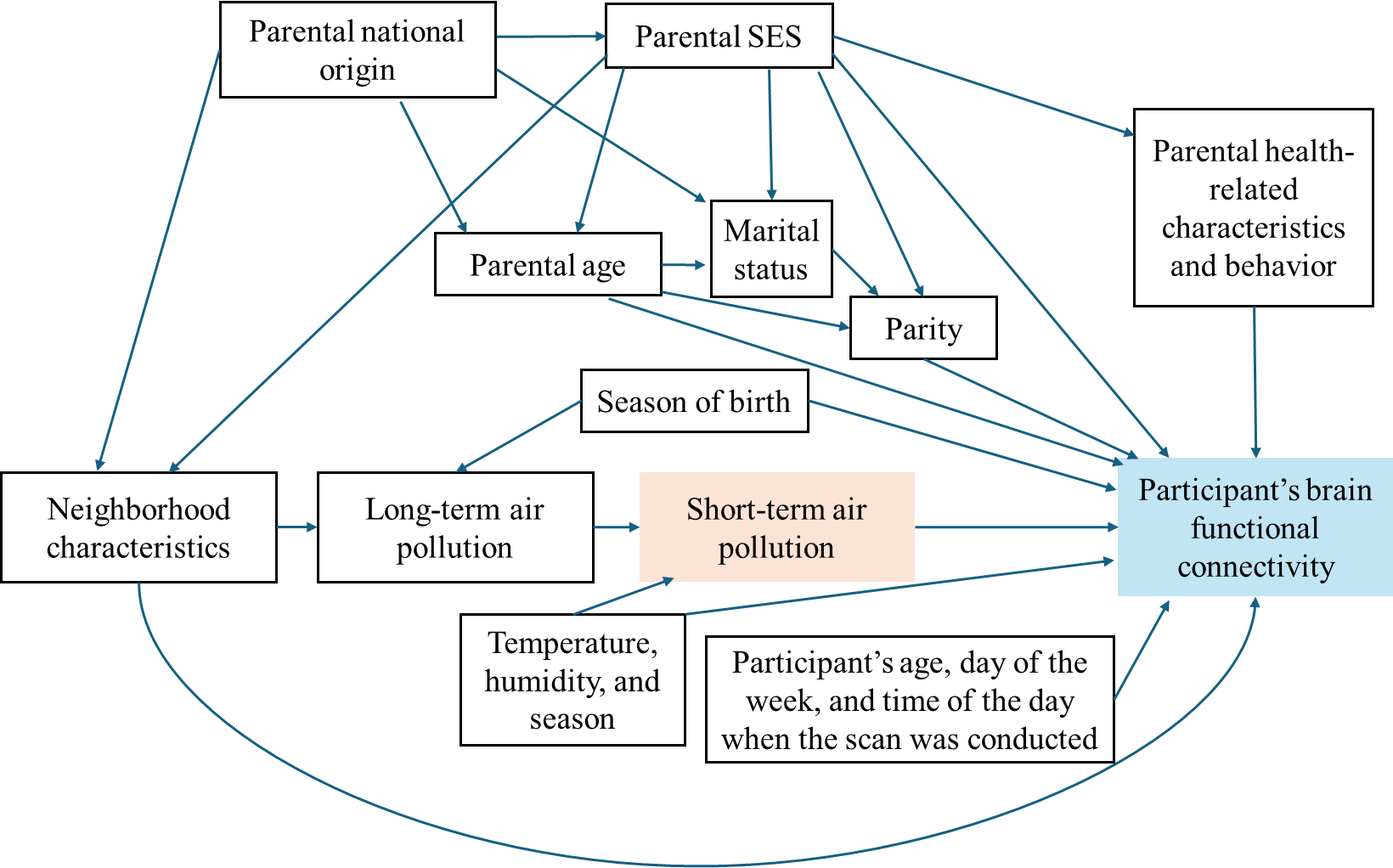


eAppendix 4. Directed Acyclic Graph

Short-term air pollution, temperature, and humidity during the week before the MRI scans. Neighborhood characteristics included residential surrounding greenness and socioeconomic status of the neighborhood. Parental SES included maternal IQ, maternal education, and monthly household income. Parental health-related characteristics and behavior included maternal pre-pregnancy body mass index, maternal smoking, maternal alcohol consumption, and maternal folic acid supplement during pregnancy.

Age-10 visit (home) Age-14 visit (home)


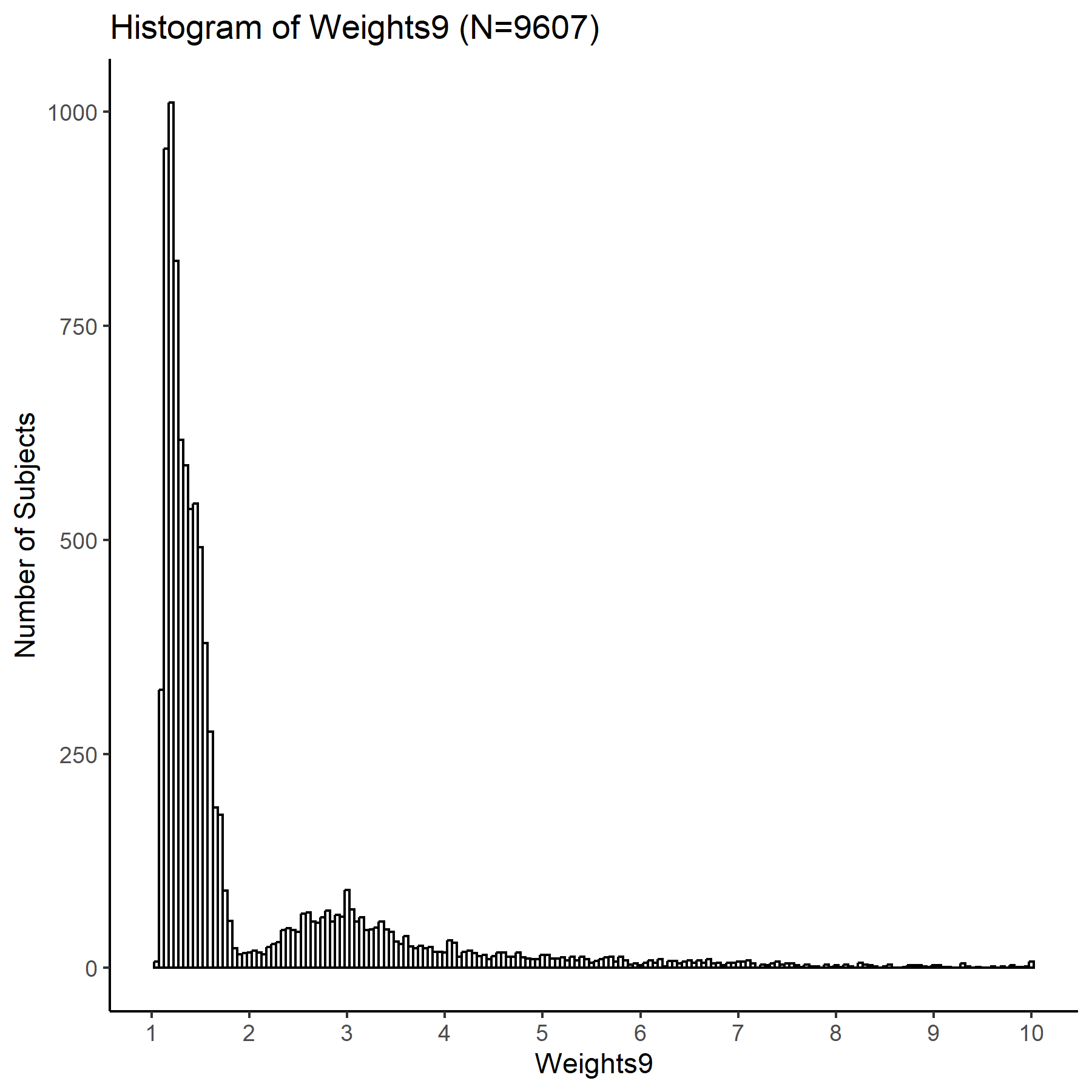

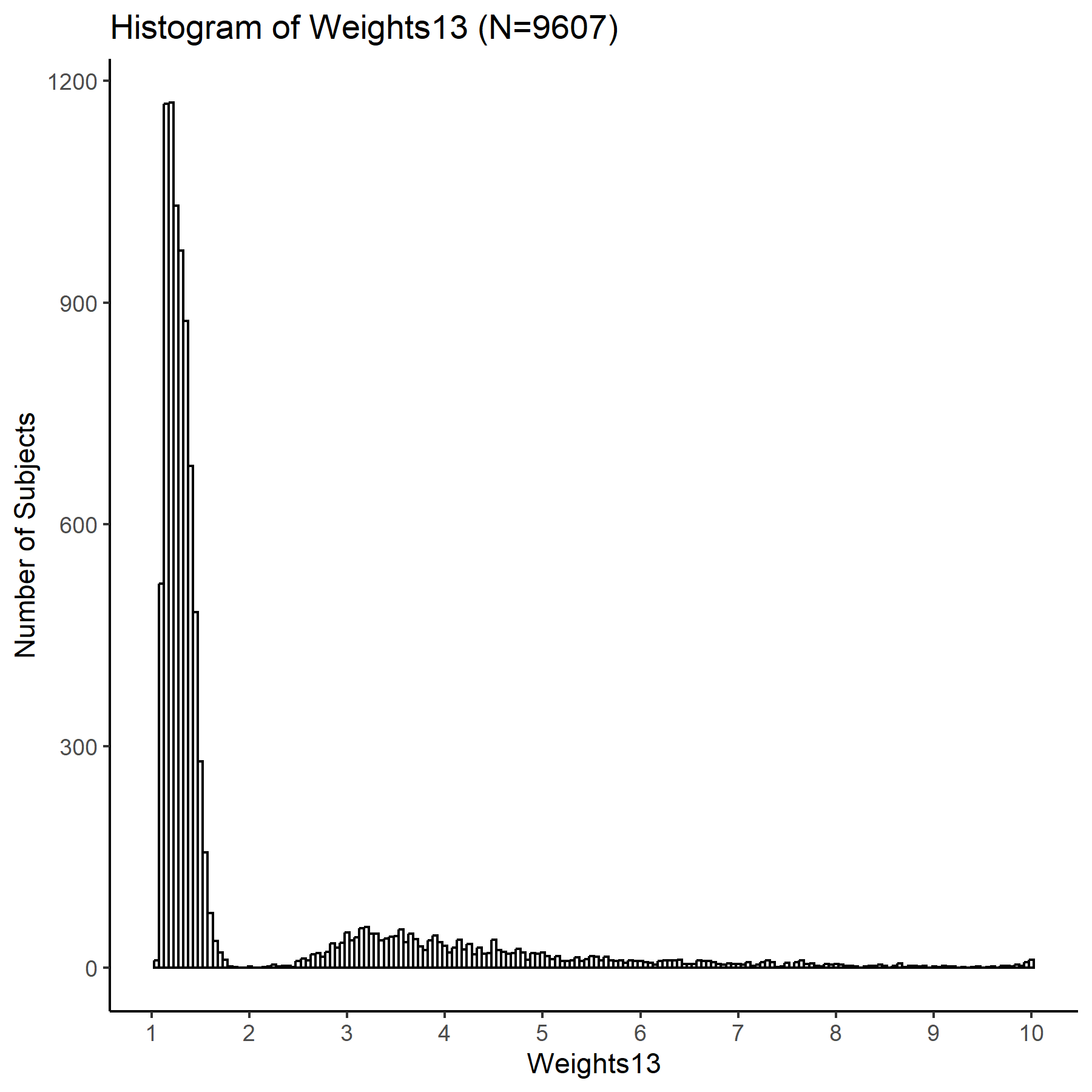


Age-10 visit (school) Age-14 visit (school)


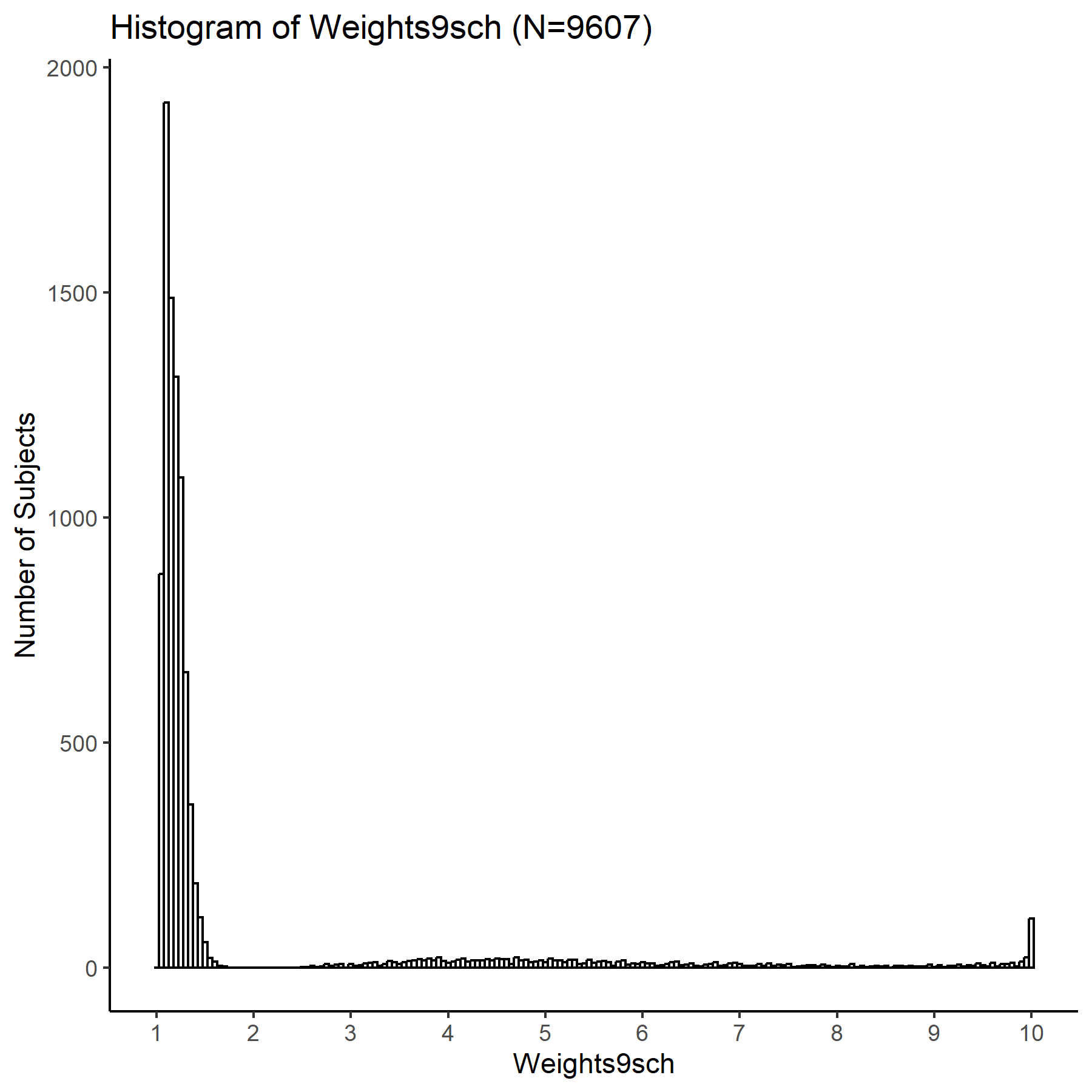

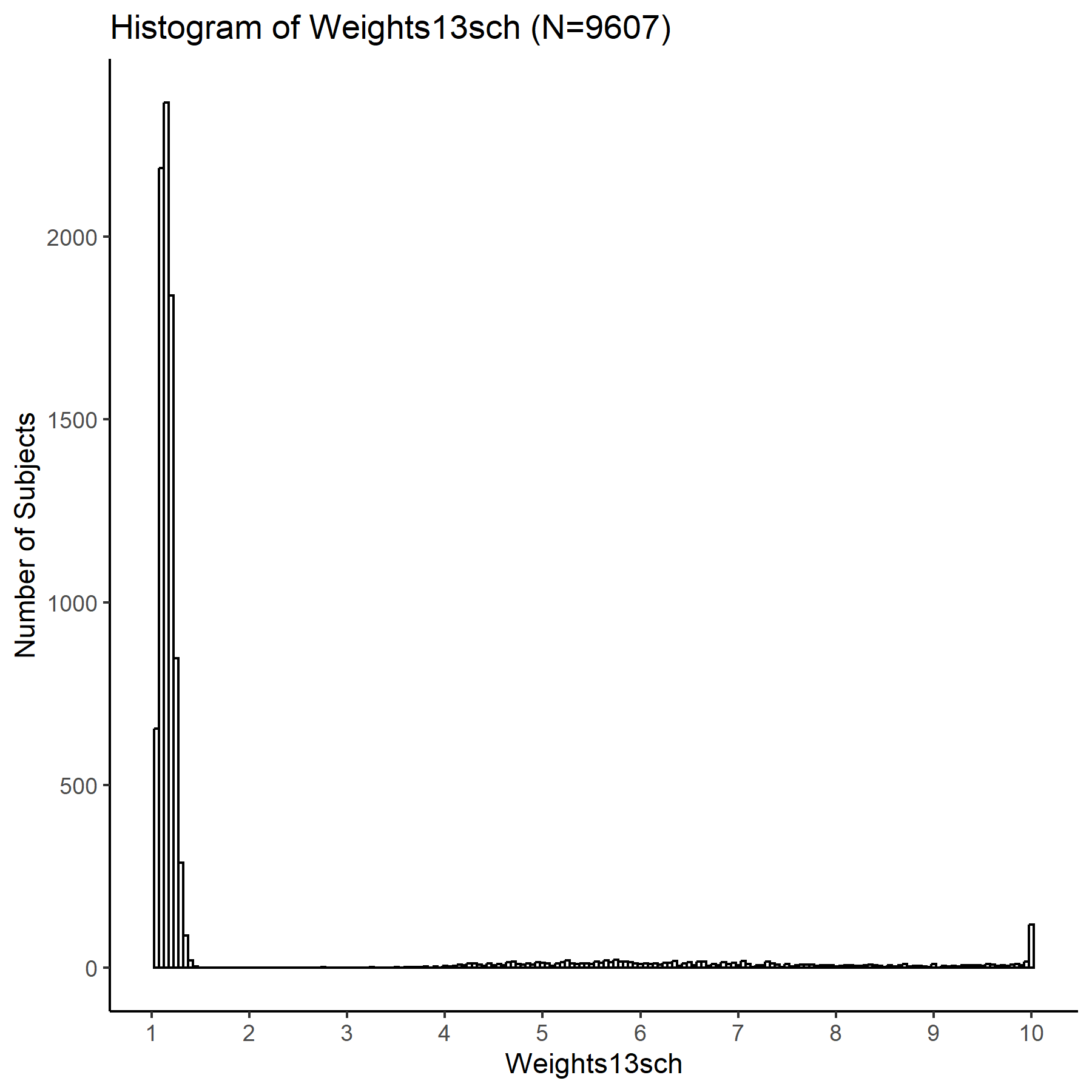


eAppendix 5. Distribution of the inverse probability weights (IPW)

First, we performed multiple imputation of missing values in covariates for the initial population (n = 9,607) using chained equations to generate 25 complete datasets. In each of these datasets, we performed the Covariate Balancing Propensity Score (CBPS) method for each visit and location (home/school) using the following predictors: participant’s sex, birth season, maternal IQ, parental national origin, parental pre-pregnancy body mass index, and covariates during pregnancy, including parental ages, maternal education, monthly household income, maternal smoking, maternal alcohol consumption, maternal folic acid supplement, maternal parity, marital status, residential surrounding greenness, socioeconomic status of the neighborhood, NO_2_ and PM_2.5_ exposure. We calculated the inverse of the probability of participation for each imputed dataset. To reduce the influence of extreme values, we winsorized these weights to a maximum of 10. We used the mean values across all 25 imputed datasets as weights for each participant in the analyses, so that results would be representative of the initial populations.


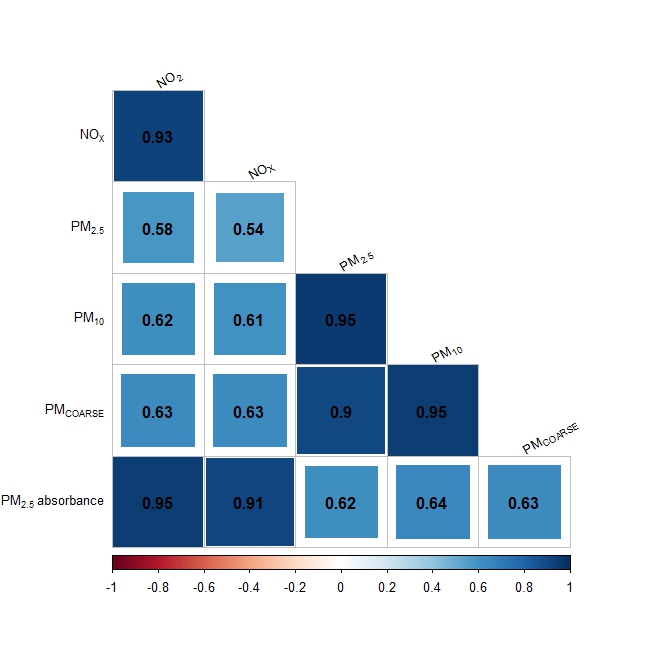


# eAppendix 6. Spearman correlations between pollutant levels at home during the week before the MRI visits

Abbreviations: NO_X_, nitrogen oxides in μg/m^3^; NO_2_, nitrogen dioxide in μg/m^3^; PM, particulate matter with different aerodynamic diameters: less than 10 μm (PM_10_) in μg/m^3^; between 10 μm and 2.5 μm (PM_COARSE_) in μg/m^3^; less than 2.5 μm (PM_2.5_) in μg/m^3^; PM_2.5_ absorbance, absorbance of PM_2.5_ filters in 10^-5^m^-1^.


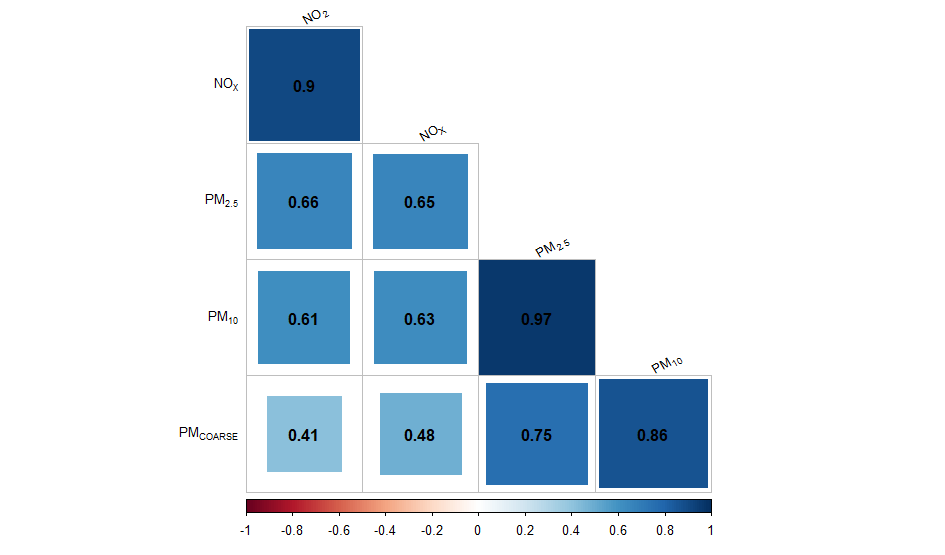


# eAppendix 7. Spearman correlations between pollutant levels at school during the week before the MRI visits

Abbreviations: NO_X_, nitrogen oxides in μg/m^3^; NO_2_, nitrogen dioxide in μg/m^3^; PM, particulate matter with different aerodynamic diameters: less than 10 μm (PM_10_) in μg/m^3^; between 10 μm and 2.5 μm (PM_COARSE_) in μg/m^3^; less than 2.5 μm (PM_2.5_) in μg/m^3^.

# eAppendix 8. Short-term associations (beta and 95% confidence interval) between exposure to each air pollutant at home (n = 3,608) and school (n = 2,305) and mean dwell time in the 5 states, adjusted for time-varying covariates only

| Exposures | | State 1 | State 2 | State 3 | State 4 | State 5 |
| --- | --- | --- | --- | --- | --- | --- |
| NO_2_ (Δ 10 μg/m^3^) | Home | 0.007 (-0.022; 0.037) | 0.015 (-0.017; 0.048) | -0.003 (-0.035; 0.030) | -0.034 (-0.066; -0.001)* | -0.018 (-0.049; 0.012) |
|  | School | 0.003 (-0.034; 0.040) | 0.005 (-0.036; 0.046) | -0.021 (-0.061; 0.019) | -0.010 (-0.051; 0.031) | 0.025 (-0.014; 0.064) |
| NO_X_ (Δ 20 μg/m^3^) | Home | 0.016 (-0.007; 0.038) | 0.017 (-0.008; 0.042) | 0.003 (-0.022; 0.027) | **-0.033 (-0.057; -0.008)*** | -0.017 (-0.041; 0.006) |
|  | School | 0.004 (-0.026; 0.034) | -0.007 (-0.041; 0.027) | -0.026 (-0.059; 0.007) | -0.001 (-0.034; 0.033) | 0.016 (-0.016; 0.047) |
| PM_2.5_ (Δ 5 μg/m^3^) | Home | 0.006 (-0.022; 0.033) | 0.003 (-0.027; 0.034) | -0.003 (-0.034; 0.027) | 0.001 (-0.029; 0.031) | -0.000 (-0.029; 0.029) |
|  | School | 0.001 (-0.028; 0.031) | -0.010 (-0.043; 0.024) | -0.007 (-0.039; 0.025) | 0.030 (-0.003; 0.063) | 0.009 (-0.022; 0.040) |
| PM_10_ (Δ 10 μg/m^3^) | Home | 0.009 (-0.026; 0.044) | 0.014 (-0.025; 0.053) | 0.004 (-0.034; 0.043) | -0.010 (-0.048; 0.029) | -0.008 (-0.045; 0.029) |
|  | School | 0.005 (-0.035; 0.044) | -0.010 (-0.054; 0.034) | -0.010 (-0.053; 0.032) | 0.036 (-0.008; 0.079) | 0.014 (-0.027; 0.055) |
| PM_COARSE_ (Δ 5 μg/m^3^) | Home | 0.016 (-0.030; 0.063) | 0.017 (-0.035; 0.069) | 0.004 (-0.047; 0.056) | -0.016 (-0.067; 0.035) | -0.019 (-0.068; 0.030) |
|  | School | 0.015 (-0.041; 0.070) | 0.001 (-0.061; 0.063) | 0.001 (-0.059; 0.061) | 0.034 (-0.028; 0.095) | 0.038 (-0.021; 0.096) |
| PM_2.5_ absorbance (10^-5^m^-1^) | Home | 0.035 (-0.029; 0.099) | 0.032 (-0.039; 0.103) | -0.015 (-0.085; 0.055) | -0.076 (-0.146; -0.006)* | -0.009 (-0.076; 0.058) |

Abbreviations: NO_X_, nitrogen oxides; NO_2_, nitrogen dioxide; PM, particulate matter with different aerodynamic diameters: less than 10 μm (PM_10_); between 10 μm and 2.5 μm (PM_COARSE_); less than 2.5 μm (PM_2.5_); PM_2.5_ absorbance, absorbance of PM_2.5_ filters. State 1, “drowsy”; State 2, default-mode/sensorimotor modularized; State 3, default-mode network modularized; State 4, non-modularized; State 5, partially modularized. Linear mixed effects models performed independently for each exposure and outcome, adjusted for participant’s age at MRI assessments, temperature, humidity, season, day of the week, and time of the day when the scan was conducted. * p value <0.05; bold: significant after multiple testing correction, new p-value 0.01.

# eAppendix 9. Short-term associations (beta and 95% confidence interval) between exposure to each air pollutant at home (n = 3,608) and school (n = 2,305) and mean dwell time in the 5 states, adjusted for long-term exposure

| Exposures | | State 1 | State 2 | State 3 | State 4 | State 5 |
| --- | --- | --- | --- | --- | --- | --- |
| NO_2_ (Δ 10 μg/m^3^) | Home | -0.010 (-0.044; 0.023) | 0.010 (-0.027; 0.048) | -0.009 (-0.046; 0.028) | -0.021 (-0.058; 0.016) | 0.012 (-0.023; 0.048) |
|  | School | -0.002 (-0.039; 0.035) | -0.000 (-0.042; 0.042) | -0.024 (-0.064; 0.017) | -0.004 (-0.046; 0.038) | 0.034 (-0.005; 0.074) |
| NO_X_ (Δ 20 μg/m^3^) | Home | 0.007 (-0.020; 0.034) | 0.010 (-0.019; 0.040) | -0.005 (-0.034; 0.024) | -0.025 (-0.054; 0.005) | 0.002 (-0.026; 0.030) |
|  | School | -0.002 (-0.033; 0.028) | -0.010 (-0.044; 0.025) | -0.027 (-0.060; 0.006) | 0.004 (-0.030; 0.038) | 0.024 (-0.008; 0.056) |
| PM_2.5_ (Δ 5 μg/m^3^) | Home | 0.003 (-0.025; 0.030) | 0.004 (-0.027; 0.035) | -0.002 (-0.033; 0.028) | 0.003 (-0.027; 0.033) | 0.002 (-0.027; 0.031) |
|  | School | -0.002 (-0.032; 0.028) | -0.008 (-0.042; 0.025) | -0.005 (-0.038; 0.027) | 0.031 (-0.002; 0.064) | 0.010 (-0.021; 0.042) |
| PM_10_ (Δ 10 μg/m^3^) | Home | 0.002 (-0.034; 0.038) | 0.012 (-0.028; 0.052) | 0.003 (-0.036; 0.043) | -0.003 (-0.042; 0.036) | -0.002 (-0.039; 0.036) |
|  | School | 0.001 (-0.038; 0.040) | -0.008 (-0.053; 0.036) | -0.009 (-0.052; 0.034) | 0.038 (-0.006; 0.082) | 0.016 (-0.026; 0.058) |
| PM_COARSE_ (Δ 5 μg/m^3^) | Home | -0.000 (-0.049; 0.048) | 0.011 (-0.043; 0.065) | 0.003 (-0.050; 0.057) | 0.003 (-0.051; 0.056) | 0.003 (-0.048; 0.054) |
|  | School | 0.005 (-0.051; 0.061) | -0.000 (-0.063; 0.063) | 0.007 (-0.055; 0.068) | 0.041 (-0.021; 0.104) | 0.049 (-0.010; 0.109) |
| PM_2.5_ absorbance (10^-5^m^-1^) | Home | 0.016 (-0.052; 0.083) | 0.023 (-0.052; 0.099) | -0.027 (-0.102; 0.047) | -0.056 (-0.130; 0.019) | 0.024 (-0.048; 0.095) |

Abbreviations: NO_X_, nitrogen oxides; NO_2_, nitrogen dioxide; PM, particulate matter with different aerodynamic diameters: less than 10 μm (PM_10_); between 10 μm and 2.5 μm (PM_COARSE_); less than 2.5 μm (PM_2.5_); PM_2.5_ absorbance, absorbance of PM_2.5_ filters. State 1, “drowsy”; State 2, default-mode/sensorimotor modularized; State 3, default-mode network modularized; State 4, non-modularized; State 5, partially modularized. Linear mixed effects models performed independently for each exposure and outcome, adjusted for participant’s age at MRI assessments, temperature, humidity, season, day of the week, time of the day when the scan was conducted, season of birth, maternal IQ, maternal national origin, maternal pre-pregnancy body mass index, and covariates during pregnancy, such as parental ages, maternal education, monthly household income, maternal smoking, maternal alcohol consumption, maternal folic acid supplement, residential surrounding greenness, socioeconomic status of the neighborhood, maternal parity, marital status, and long-term exposure to each specific pollutant at home.

# eAppendix 10. Short-term associations (beta and 95% confidence interval) between exposure to each air pollutant at home (n = 2,475) and school (n = 1,498) and mean dwell time in the 5 states, age-10 years visit

| Exposures | | State 1 | State 2 | State 3 | State 4 | State 5 |
| --- | --- | --- | --- | --- | --- | --- |
| NO_2_ (Δ 10 μg/m^3^) | Home | -0.034 (-0.078; 0.010) | 0.026 (-0.023; 0.074) | 0.014 (-0.030; 0.058) | -0.030 (-0.080; 0.019) | -0.007 (-0.056; 0.041) |
|  | School | -0.053 (-0.104; -0.001)* | 0.023 (-0.037; 0.083) | -0.024 (-0.078; 0.031) | -0.006 (-0.067; 0.055) | 0.038 (-0.024; 0.099) |
| NO_X_ (Δ 20 μg/m^3^) | Home | -0.014 (-0.048; 0.019) | 0.027 (-0.012; 0.066) | 0.017 (-0.018; 0.053) | -0.034 (-0.073; 0.005) | -0.013 (-0.053; 0.026) |
|  | School | -0.035 (-0.071; 0.002) | 0.007 (-0.039; 0.052) | -0.022 (-0.064; 0.019) | -0.003 (-0.048; 0.042) | 0.021 (-0.027; 0.068) |
| PM_2.5_ (Δ 5 μg/m^3^) | Home | -0.020 (-0.059; 0.018) | -0.010 (-0.052; 0.033) | -0.002 (-0.046; 0.042) | 0.026 (-0.021; 0.072) | -0.017 (-0.058; 0.025) |
|  | School | -0.015 (-0.053; 0.022) | -0.015 (-0.057; 0.026) | -0.023 (-0.066; 0.021) | 0.046 (0.000; 0.092)* | -0.014 (-0.054; 0.026) |
| PM_10_ (Δ 10 μg/m^3^) | Home | -0.021 (-0.069; 0.026) | -0.006 (-0.059; 0.048) | 0.002 (-0.052; 0.057) | 0.021 (-0.038; 0.079) | -0.026 (-0.079; 0.026) |
|  | School | -0.017 (-0.067; 0.034) | -0.020 (-0.076; 0.036) | -0.035 (-0.093; 0.023) | 0.058 (-0.003; 0.118) | -0.016 (-0.070; 0.038) |
| PM_COARSE_ (Δ 5 μg/m^3^) | Home | -0.019 (-0.084; 0.045) | -0.007 (-0.077; 0.062) | 0.003 (-0.068; 0.075) | 0.015 (-0.063; 0.093) | -0.033 (-0.102; 0.037) |
|  | School | -0.030 (-0.107; 0.047) | -0.052 (-0.135; 0.030) | -0.051 (-0.137; 0.036) | 0.088 (-0.003; 0.178) | 0.018 (-0.062; 0.098) |
| PM_2.5_ absorbance (10^-5^m^-1^) | Home | -0.070 (-0.174; 0.033) | 0.050 (-0.067; 0.167) | 0.021 (-0.084; 0.126) | -0.082 (-0.202; 0.038) | 0.029 (-0.089; 0.148) |

Abbreviations: NO_X_, nitrogen oxides; NO_2_, nitrogen dioxide; PM, particulate matter with different aerodynamic diameters: less than 10 μm (PM_10_); between 10 μm and 2.5 μm (PM_COARSE_); less than 2.5 μm (PM_2.5_); PM_2.5_ absorbance, absorbance of PM_2.5_ filters. State 1, “drowsy”; State 2, default-mode/sensorimotor modularized; State 3, default-mode network modularized; State 4, non-modularized; State 5, partially modularized. Linear mixed effects models performed independently for each exposure and outcome, adjusted for participant’s age at MRI assessments, temperature, humidity, season, day of the week, time of the day when the scan was conducted, season of birth, maternal IQ, maternal national origin, maternal pre-pregnancy body mass index, and covariates during pregnancy, such as parental ages, maternal education, monthly household income, maternal smoking, maternal alcohol consumption, maternal folic acid supplement, residential surrounding greenness, socioeconomic status of the neighborhood, maternal parity, and marital status. * p value <0.05 (non-significant after multiple testing correction, new p-value 0.01).

# eAppendix 11. Short-term associations (beta and 95% confidence interval) between exposure to each air pollutant at home (n = 2,121) and school (n = 1,312) and mean dwell time in the 5 states, age-14 years visit

| Exposures | | State 1 | State 2 | State 3 | State 4 | State 5 |
| --- | --- | --- | --- | --- | --- | --- |
| NO_2_ (Δ 10 μg/m^3^) | Home | -0.009 (-0.055; 0.037) | 0.029 (-0.020; 0.077) | 0.014 (-0.035; 0.062) | -0.029 (-0.075; 0.018) | -0.005 (-0.052; 0.042) |
|  | School | 0.022 (-0.040; 0.085) | 0.005 (-0.063; 0.072) | 0.001 (-0.063; 0.065) | -0.012 (-0.075; 0.052) | 0.029 (-0.037; 0.096) |
| NO_X_ (Δ 20 μg/m^3^) | Home | 0.007 (-0.026; 0.040) | 0.025 (-0.010; 0.060) | 0.010 (-0.026; 0.045) | -0.030 (-0.063; 0.003) | -0.007 (-0.040; 0.026) |
|  | School | 0.025 (-0.032; 0.083) | -0.006 (-0.068; 0.055) | -0.012 (-0.070; 0.047) | -0.001 (-0.061; 0.060) | 0.034 (-0.029; 0.096) |
| PM_2.5_ (Δ 5 μg/m^3^) | Home | 0.012 (-0.036; 0.060) | 0.012 (-0.036; 0.060) | 0.014 (-0.032; 0.060) | -0.009 (-0.055; 0.037) | 0.016 (-0.038; 0.070) |
|  | School | 0.017 (-0.038; 0.072) | 0.003 (-0.054; 0.060) | 0.029 (-0.026; 0.083) | 0.022 (-0.032; 0.077) | 0.042 (-0.017; 0.100) |
| PM_10_ (Δ 10 μg/m^3^) | Home | 0.014 (-0.049; 0.076) | 0.040 (-0.021; 0.102) | 0.028 (-0.033; 0.088) | -0.030 (-0.091; 0.030) | 0.019 (-0.051; 0.089) |
|  | School | 0.020 (-0.052; 0.092) | 0.015 (-0.061; 0.091) | 0.037 (-0.035; 0.109) | 0.023 (-0.050; 0.095) | 0.060 (-0.016; 0.136) |
| PM_COARSE_ (Δ 5 μg/m^3^) | Home | -0.002 (-0.085; 0.082) | 0.041 (-0.042; 0.124) | 0.038 (-0.043; 0.119) | -0.015 (-0.096; 0.066) | 0.022 (-0.065; 0.110) |
|  | School | 0.025 (-0.073; 0.122) | 0.064 (-0.042; 0.169) | 0.071 (-0.025; 0.168) | 0.011 (-0.090; 0.113) | 0.097 (-0.006; 0.201) |
| PM_2.5_ absorbance (10^-5^m^-1^) | Home | 0.012 (-0.082; 0.105) | 0.040 (-0.061; 0.141) | 0.021 (-0.076; 0.118) | -0.059 (-0.155; 0.037) | 0.004 (-0.092; 0.100) |

Abbreviations: NO_X_, nitrogen oxides; NO_2_, nitrogen dioxide; PM, particulate matter with different aerodynamic diameters: less than 10 μm (PM_10_); between 10 μm and 2.5 μm (PM_COARSE_); less than 2.5 μm (PM_2.5_); PM_2.5_ absorbance, absorbance of PM_2.5_ filters. State 1, “drowsy”; State 2, default-mode/sensorimotor modularized; State 3, default-mode network modularized; State 4, non-modularized; State 5, partially modularized. Linear mixed effects models performed independently for each exposure and outcome, adjusted for participant’s age at MRI assessments, temperature, humidity, season, day of the week, time of the day when the scan was conducted, season of birth, maternal IQ, maternal national origin, maternal pre-pregnancy body mass index, and covariates during pregnancy, such as parental ages, maternal education, monthly household income, maternal smoking, maternal alcohol consumption, maternal folic acid supplement, residential surrounding greenness, socioeconomic status of the neighborhood, maternal parity, and marital status.

# References

1. White T, Muetzel RL, El Marroun H, et al. Paediatric population neuroimaging and the Generation R Study: the second wave. *Eur J Epidemiol*. Jan 2018;33(1):99-125.

2. Esteban O, Markiewicz CJ, Blair RW, et al. fMRIPrep: a robust preprocessing pipeline for functional MRI. *Nat Methods*. Jan 2019;16(1):111-116.
